# Effect of hybrid FES exercise on body composition during the sub-acute phase of spinal cord injury

**DOI:** 10.1371/journal.pone.0262864

**Published:** 2022-01-24

**Authors:** Khashayar Afshari, Erin D. Ozturk, Brandon Yates, Glen Picard, J. Andrew Taylor

**Affiliations:** 1 Department of Physical Medicine and Rehabilitation, Harvard Medical School, Boston, MA, United States of America; 2 Cardiovascular Research Laboratory, Spaulding Hospital Cambridge, Cambridge, MA, United States of America; 3 Spaulding Research Institute, Charlestown, MA, United States of America; The University of British Columbia, CANADA

## Abstract

**Objectives:**

To determine the Effect of Hybrid functional electrically stimulated (FES) Exercise on Body Composition during the Sub-acute Phase of Spinal Cord Injury (SCI).

**Design:**

Randomized Clinical Trial.

**Setting:**

Rehabilitation Hospital.

**Participants:**

Patients within sub-acute phase (3–24 months) of SCI.

**Interventions:**

We investigated if high-intensity exercise training via the addition of functional electrically stimulated (FES) leg muscles, provides sufficient stimulus to mitigate against body composition changes in the sub-acute phase after SCI.

**Main outcome measures:**

We explored potential effects of FES row training (FESRT) on body fat gain, lean mass loss, and cardiometabolic parameters and compared the effects of 6-month of FESRT (n = 18) to standard of care (SOC, n = 13). Those in SOC were crossed over to FESRT.

**Results:**

FESRT resulted in greater exercise capacity and a tendency for lesser total body fat accumulation with a significant increase in total and leg lean mass (p<0.05). In addition pelvis and total bone mineral density declines were significantly less (p<0.05). Compared to SOC, FESRT did not lead to any significant difference in insulin sensitivity or serum lipids. However, HbA1C levels were significantly decreased in SOC participants who crossed over to 6-month FESRT.

**Conclusion:**

FESRT early after SCI provides a sufficient stimulus to mitigate against detrimental body composition changes. This may lead to prevention of losses in lean mass, including bone.

## Introduction

Body composition is dramatically impacted by spinal cord injury (SCI) [1, 2]. Those with SCI have higher fat mass, greater body fat percentage, and increased visceral adiposity. Moreover, compared to able-bodied individuals, those with chronic SCI have decreased total and regional lean mass [3–6]. These changes in body composition are major contributors to high prevalence of insulin resistance, glucose intolerance, and dyslipidemia in chronic SCI [7]. Indeed, >40% of those with chronic SCI have metabolic syndrome [3].

Body composition changes occur rapidly. Castro et al. have shown that skeletal muscle protein degradation starts within the first six months after SCI [1]. Within the first twelve months after injury, a substantial increase in body fat mass, body fat percentage, and visceral adipose tissue occurs, accompanied by decreases in total and sub-lesional lean mass with decreased bone mineral content [1, 2, 4, 8, 9]. The sub-lesional lean mass and bone decreases are rapid and most severe in the first three years, but with time become progressively worse [4]. However, exercise may mitigate these undesirable changes in body composition [10]. Exercise can reduce body fat and visceral adipose, enhance muscle and lean mass, and improve serum lipids and insulin sensitivity. These effects vary based on the type of exercise, as well as the known effects of exercise frequency, duration, and intensity [11–14].

Exercise for those with SCI is mainly limited to unaffected muscles. Arm crank endurance training improves cardiovascular fitness when performed at high intensities, but with limited increases in upper limb muscle [14–16] whereas resistance training can enhance upper body lean mass [17, 18]. Circuit training involving both exercises substantially increases peak oxygen uptake (VO_2peak_) and muscle strength, and counters dyslipidemia and reduces shoulder pain [17, 19]. However, individuals with intact lower motor neurons may benefit from addition of the paralyzed muscles to exercise via functional electrical stimulation (FES) [20]. This provides the ability for assisted cycling, rowing, or ambulation training. Moreover, hybrid exercises involving both upper and lower extremities require greater muscle mass and hence result in improved cardiovascular fitness and lean mass. Among these hybrid exercises, FES-rowing provides a coordinated, full body exercise that may be more beneficial than the limited muscle mass of conventional arm cycling exercise [17, 21, 22].

Studies in able-bodied individuals indicate that improved body composition [23, 24] and cardiovascular fitness [25, 26] directly relate to exercise intensity. In SCI, higher intensity exercise is more effective in increasing peak oxygen uptake [27–29] and may be associated with higher resultant lean mass and bone density [16, 30, 31]. Indeed, hybrid FES exercise at higher exercise intensities holds promise to mitigate adiposity, sarcopenia, and metabolic dysregulation [32–35]. Although effectiveness has been evaluated primarily in chronic SCI (>~2 years), early interventions employing FES within 3–6 months after injury may increase lean mass and potentially impact glucose levels and carbohydrate metabolism [30]. However, there are scarce studies on effective exercises early after SCI and none have compared high-intensity training with standard of care practices—upper extremity training only or no exercise.

As part of a larger clinical trial on FES-RT effectiveness [36–41], the current work sought to determine if high-intensity exercise training via the addition of stimulated leg muscles provides a sufficient stimulus to mitigate against body fat gain and lean mass loss in the sub-acute 24 months after SCI. We also explored potential effects on cardiometabolic parameters—insulin sensitivity and blood lipids. We compared the effects of 6 months of FES row training (FESRT) to standard of care (SOC), represented by arms-only exercise (AO) and waitlist (no exercise, WL).

## Methods

### Participants

**Fig 1:**
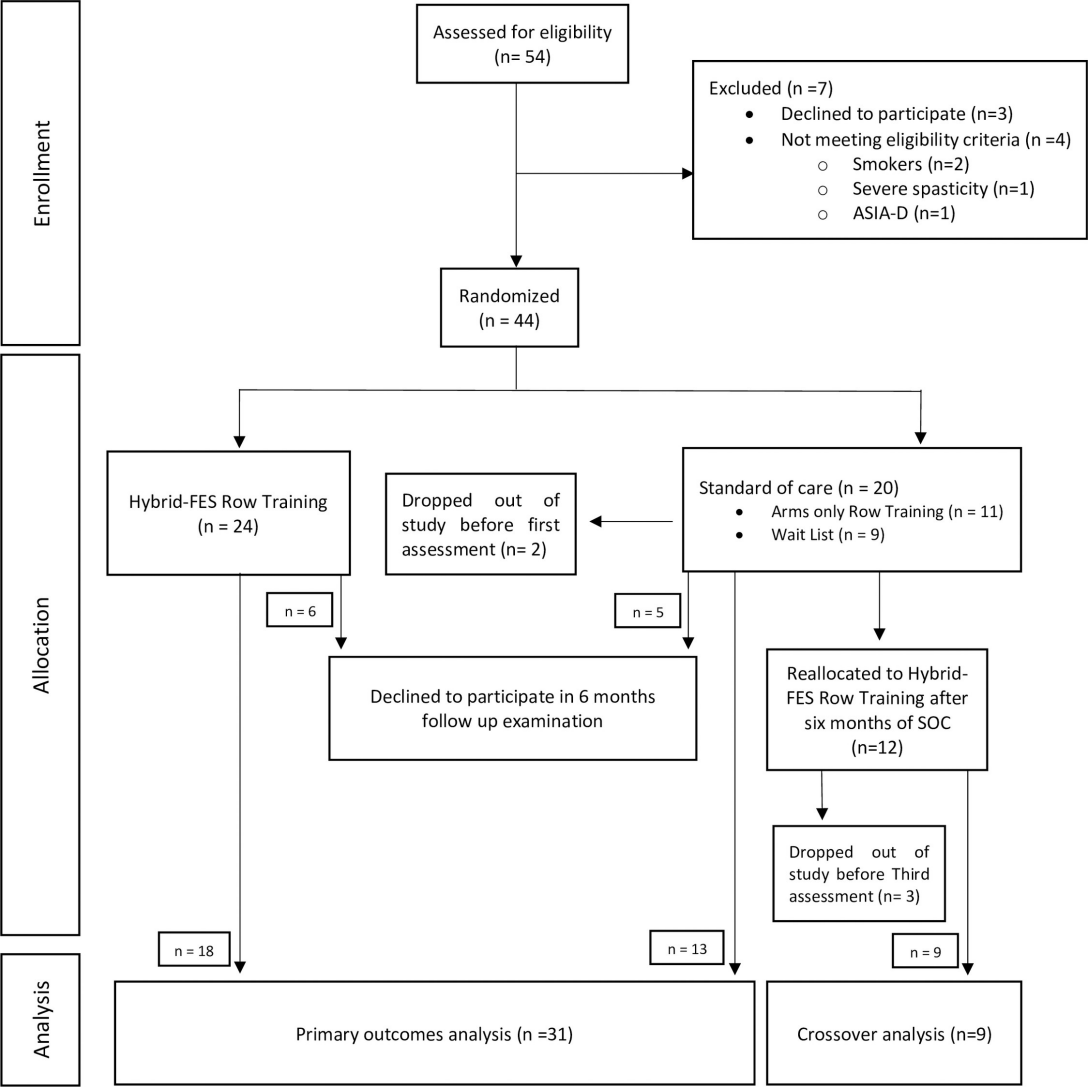
Consort Flow diagram. Flow diagram showing participants flow through the randomized controlled trial and primary and crossover results analysis.

Participants were primarily recruited from the Spaulding Rehabilitation Network. They were approached during inpatient rehabilitation and provided with information and consent forms to gauge their interest. Subsequently, participants were contacted and recruited based on the inclusion / exclusion criteria. Individuals 18–40 years within 24 months of SCI were enrolled between 2013 to 2019. We restricted our age range to those under 40 to avoid the confounds of age-related sarcopenia. Forty-four of 51 eligible cases were randomized to 6 months of FESRT or SOC. Of these, 31 patients completed the study (FESRT, N = 18; SOC, N = 13: 7 in AO and 6 in WL). The two later groups correspond to standard of care, in which what most participants do is aerobic exercise or arms only row training. After six months, those in SOC crossed over to 6 months of FESRT with 9 completing training. All training doses (frequency, time) where chosen based on ACSM guidelines [42]. Procedures were approved by the Spaulding Rehabilitation Hospital Institutional Review Board and written informed consent was obtained (Trial Registration NCT02139436). A CONSORT diagram is shown in Fig 1. Outcome measures were obtained at baseline, 6 months after intervention, and 6 months after crossover.

Participants were SCI outpatients medically stable with no treatment for DVT, no orthostatic intolerance, and no spinal or weight-bearing precautions associated with a healing, long-bone fracture. According to the inclusion criteria, participants’ neurological level of injury was between C5 to T12 and American Spinal Injury Association (ASIA) impairment scale was A, B, or C. All individuals had body mass index (BMI) within normal to overweight range. All included participants were wheelchair users who were able to follow directions, perform rowing and their leg muscles were evaluated as responsive to electrical stimulation. No adverse events occurred over the course of the study.

### Exercise protocol

#### Functional electrical stimulation row training

Participants performed 2–3 weeks of FES strength training to allow for the capacity to perform FESRT. For this, electrodes over motor points of the quadriceps and hamstrings provided stimulus intensity to produce full knee flexion-extension. Training was performed 3x/week until participants could complete the protocol for 30 min without rest. Subsequently, FESRT exercise commenced. Exercise sessions were prescribed for 3x/week for 26 weeks.

Initial training sessions were 6 sets of FES-rowing for 5 min at 60% of VO_2_peak with a work-to-rest ratio of 2:1 and progressed to continuous exercise. A maximum FES-rowing test was performed at baseline and after 3 months of training to adjust training intensity. The goal was to achieve an exercise intensity of 70–85% of VO_2_peak for 30–40 min 3x/week. A final maximum FES-rowing test was performed at end of study.

#### Arms only rowing training

AO training did not require a period of strength training and commenced upon enrollment. Parallel to FESRT, training sessions were 3x/week for 26 weeks. Initial training sessions, intervals, duration, and intensity goals and maximum performance tests were parallel to FESRT. A maximum AO rowing test was performed at baseline, after 3 months of training to adjust training intensity, and at end of study.

#### Wait list

The WL control group was randomly allocated as a 6 month time control to receive only regular physical rehabilitation. This excluded modalities that overlapped other study interventions (i.e., FES cycling, arm crank training).

#### Cross over

After completing the six months period, participants in standard care groups of arms only training and wait list were asked to participate in FES row training, 9 of these participants crossed to FESRT and completed the six-month training period. This group of participants was considered as a cross-over group.

### Assessments

#### Exercise capacity

As described in our prior publications from the larger clinical dataset on the applied intervention of FESRT, maximal aerobic power (VO2max) [36–41] was determined from online computer assisted open circuit spirometry (ParvoMedics, Sandy, UT). Ventilation and expired O2 and CO2 were measured to determine O2 consumption (VO2), CO2 production (VCO2), respiratory exchange ratio (RER), and ventilation (VE). Expired O2 and CO2 gas fractions were measured with paramagnetic O2 and infrared CO2 analyzers. Ventilation volumes were measured via a pneumotachograph.

#### Dual x-ray absorptiometry

Dual x-ray absorptiometry (DA) is precise and reliable for total and regional body composition and well-validated against magnetic resonance imaging in those with SCI [43, 44]. A 5th generation GE Healthcare iDXA scanner was used for total and regional measurement of lean mass, fat mass, fat percentage, visceral adipose tissue (VAT), Bone Mineral Density (BMD) and Bone Mineral Content (BMC). Head BMD was used as an invariable quality control measure, since it is unaffected by the interventions.

#### Insulin sensitivity and cardiovascular health markers

Blood samples were obtained via venipuncture for fasting plasma glucose, insulin, hemoglobin A1C (HbA1C), and serum lipids. Although fasting insulin tends to correlate with insulin resistance, the homeostasis model assessment (HOMA) and the quantitative insulin check index (QUICKI) were used as more precise estimates of insulin resistance.

#### Basal metabolic rate

Basal Metabolic Rate (BMR) was derived from the Fat Free Mass alone model (23.469*Fat Free Mass (kg) + 294.330) developed and validated for persons with SCI by Nightingale and Gorgey [45].

### Statistical analysis

Participants were randomized at the ratio of 1.33:1:1 to each of the three groups of FES-RT, wait-list and arms-only-RT by a randomization plan constructed by the project statistician. The investigators had no knowledge of group assignment until the time of subject enrolment. Paired t-tests were used for comparing pre and post intervention exercise capacity where Kolmogorov-Smirnov and Shapiro-Wilk test showed a normal distribution. For comparing differences in body composition and blood indices, Analysis of Covariance (ANCOVA) was performed, adjusting the final means with baseline measures. Linear regression was performed to assess time and group interaction for each variable, indicating the difference of changes through time. All variables were assessed in cross over cases by comparing 12-month results to baseline and 6-month measurements. P value < 0.05 was considered statistically significant and p value < 0.2 with medium to large effect size was considered as tending to significant. Throughout the manuscript summary statistics are described as “mean ± standard deviation (SD)” unless otherwise specified. SPSS version 25 and STATA version 14 were used for statistical analysis.

## Results

Data were obtained from 31 individuals with a mean time since SCI of 11.1±5.2 months (Table 1). Those in AO and WL subgroups of SOC were statistically similar at baseline except for total lean mass (p = 0.03). Participants in exercise groups were adherent to the training plans. The FES-RT, AO training and cross over groups exercised for of 6.9 ± 1.95, 6.22 ± 0.94 and 5.96 ± 1.6 sessions a month with a mean time of 184.3 ± 65.6, 186 ± 30.52 and 159.38 ± 62.04 minutes which were statistically similar (respective p-values = 0.3, 0.5). FESRT significantly increased exercise capacity (p<0.001) whereas the SOC group as a whole and each subgroup demonstrated no change (AO, p = 0.35; WL, p = 0.5).

**Table 1:** Baseline features of enrolled individuals.

		FESRT (n = 18)	Wait List (n = 6)	Arms Only (n = 7)
Sex (Male n, %))		n = 15 (83%)	n = 6 (100%)	n = 7 (100%)
Age (Year, Mean±SD)		29.06±5.40	26.00±6.69	27.57±7.09
BMI (Kg/m2, Mean±SD))		24.10±4.29	25.45±2.81	23.94±3.62
Weight (Kg, Mean±SD))		74.12±13.46	82.70±9.88	81.58±17.64
ASIA Impairment Scale (n, %)	A	n = 9 (50%)	n = 4 (66.7%)	n = 5 (71.4%)
	B	n = 5 (27.8%)	n = 1 (16.15%)	n = 0
	B/C	n = 2 (11.1%)	n = 0	n = 1 (14.3%)
	C	n = 2 (11.1%)	n = 1 (16.15%)	n = 1 (14.3%)
Level of injury (level: n (%))		Cervical: 14 (73%), Thoracic: 5 (26%)	Cervical:4 (66%)	Cervical:4 (57%)
			Thoracic: 2 (33%)	Thoracic: 3 (43%)
Motor Complete (yes, n, %)		n = 14 (77.8%)	n = 5 (83%)	n = 5 (72%)

FESRT: Functional Electrical Stimulation Row Training, BMI: Body Mass Index, ASIA: American Spinal Injury Association.

**Fig 2:**
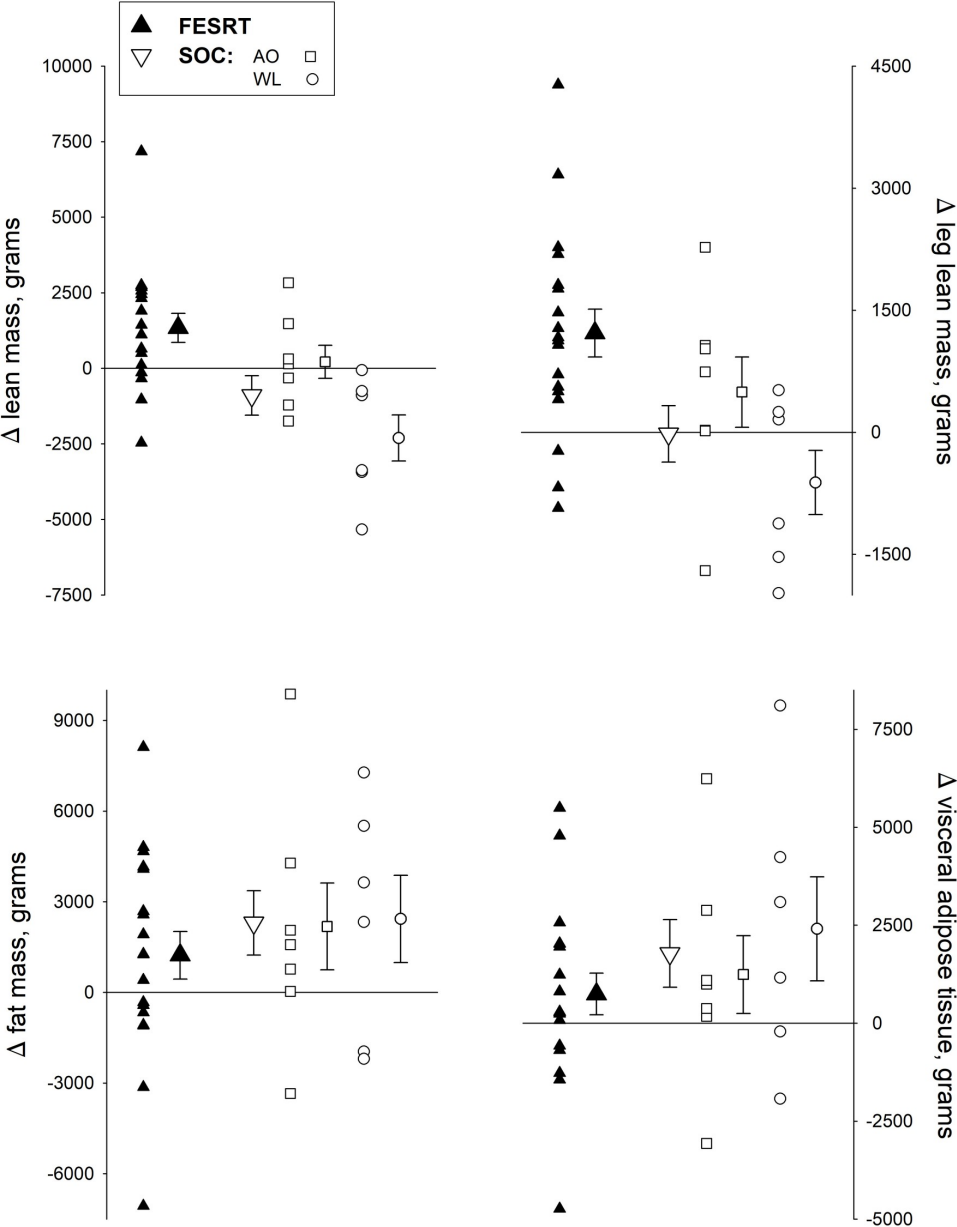
Lean and fat mass changes. Individual and group mean total lean mass, leg lean mass, fat mass and visceral adipose tissue, compared between FESRT and SOC and subgroups AO and WL. Results of regression analysis indicated significantly higher time dependent increase in total lean mass and leg lean mass in FESRT compared to SOC (p values = 0.006 and,0.012 respectively). Both FESRT and AO training were associated with a greater increase in total lean mass compared to WL (Δ3647 grams, p = 0.001 and Δ2522 grams, p = 0.017 respectively).

Compared to SOC, FESRT did not result in any significant differences in weight or BMI. Although as a group no statistically significant VAT changes were observed in FESRT subjects, VAT was decreased or relatively unchanged (<1%) in 6 subjects (33%) compared to only 3 (23%) in SOC (Fig 2). Additionally, FESRT tended to result in lesser total body fat percentage (p = 0.16, Partial Eta Squared = 0.067). This is likely due to FESRT resulting in significantly higher total lean mass (p = 0.012) and leg lean mass (p = 0.013). Additionally, regression showed a significant time and group interaction for both total lean mass and leg lean mass. This indicated that FESRT had a greater increase than SOC in total lean mass of 2289 grams (Robust Std. Err. = 781, 95% Conf. Interval: 692–3886) and in leg lean mass of 1238 grams (Robust Std. Err. = 462, 95% Conf. Interval: 292–2183). Although both FESRT and AO were associated with greater total lean mass compared to WL (3647 grams, p = 0.001 and 2522 grams, p = 0.017 respectively), only FESRT resulted in a significantly higher total lean mass compared to baseline (p = 0.002) (Fig 2). In contrast, the nine SOC subjects who completed crossover to FESRT for an additional 6 months, showed no changes in either total or leg lean mass compared to baseline or 6 months of AO or WL (p > 0.05).

**Fig 3:**
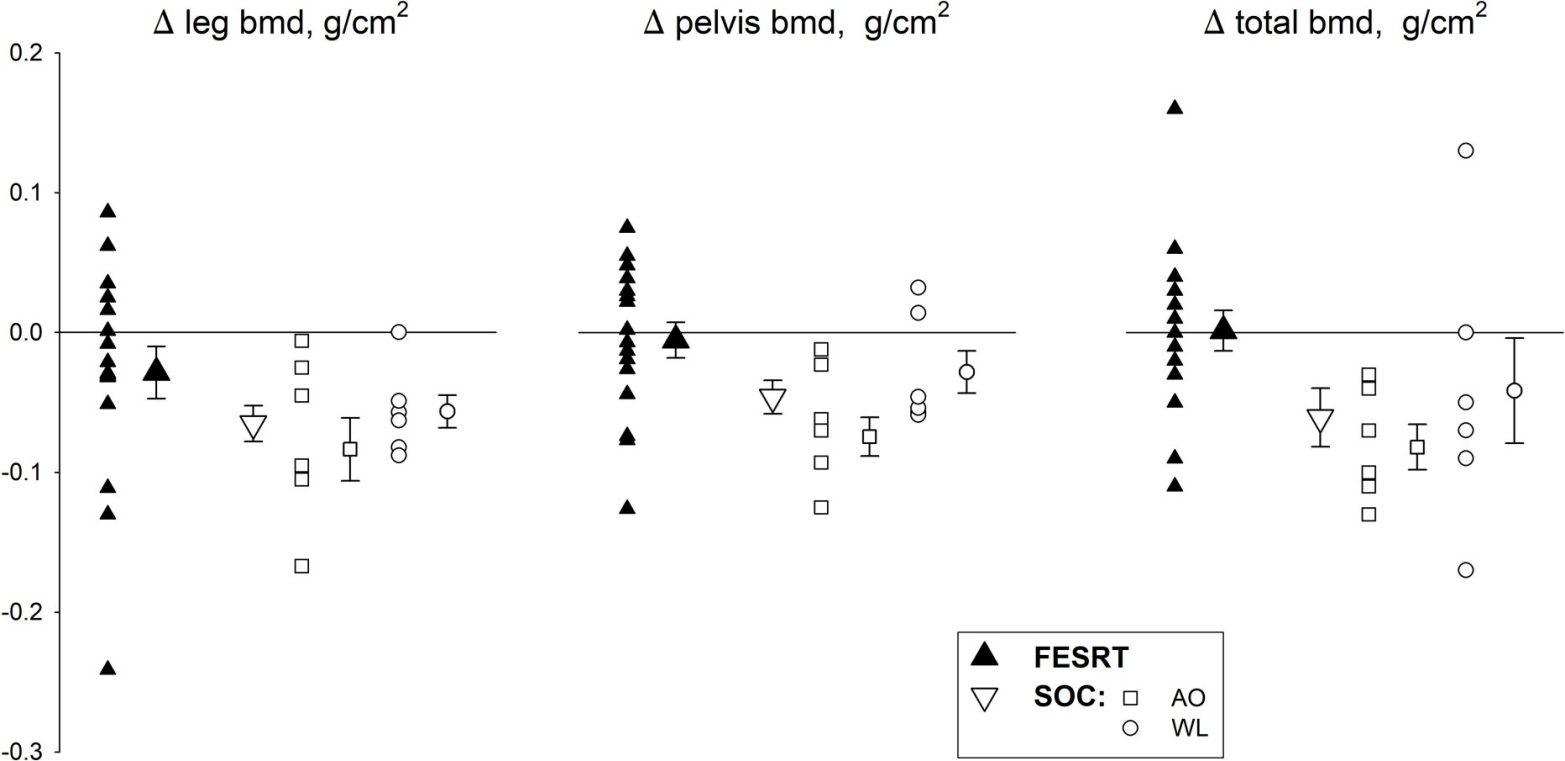
Bone density changes. Individual and group mean legs, pelvis and total bone mineral density compared between FESRT and SOC and subgroups AO and WL. Results of regression analysis indicated significantly lower time dependent decrease in total BMD and pelvis BMD of FESRT group compared to SOC (*p* = 0.039 and *p* = 0.028 respectively). Although the results for legs BMD were not statistically significant, a similar tendency was detected for this variable (*p* = 0.14, R-squared = 0.21).

Although FESRT did not result in BMC different from SOC, FESRT did have a tendency to result in greater total BMD (p = 0.13, Partial Eta Squared = 0.088) and a significant effect preventing a decrease in total BMD (p = 0.039). Additionally, FESRT resulted in higher pelvis BMD, effectively preventing losses in this region (p = 0.028). In contrast, neither BMD nor BMC in the lower legs was affected by FESRT (Fig 3). The cross over individuals demonstrated significantly decreased bone mineral content and density in all measurements except pelvis BMD at the end of 12 months, hence 6 months of FESRT appeared to help preserve bone in only this region (p<0.05).

Compared to SOC, FESRT did not lead to any significant difference in serum lipids and insulin sensitivity indices (Table 2). Although HbA1C levels were not affected within the first six months of FESRT or SOC, those who crossed over to FESRT had significantly lower HbA1C levels after training compared to baseline (p<0.001).

**Table 2:** Comparison of serum lipids, HbA1C levels, plasma glucose, plasma insulin and insulin sensitivity after six months of exercise.

Estimated Mean (SE)*	FESRT (n = 18)	SOC (n = 13)	p-value
Tg (mg/dL)	114.60 (11.28)	116.78 (13.40)	0.90
Chol (mg/dL)	172.18 (4.87)	164.51 (5.74)	0.32
LDL (mg/dL)	109.47 (4.30)	101.86 (5.28)	0.27
HDL (mg/dL)	40.65 (1.45)	39.55 (1.71)	0.63
HbA1C (%)	5.04 (0.07)	5.19 (0.08)	0.21
Fasting Plasma Glucose (mg/dL)	79.09 (1.40)	78.55 (1.66)	0.81
Fasting Plasma Insulin	4.83 (0.51)	5.71 (0.61)	0.29
HOMA-IR	0.94 (0.10)	1.14 (0.12)	0.26
QUICKI	0.38 (0.00)	0.39 (0.00)	0.88

*Based on ANCOVA analysis where final measures have been estimated by adjustments to primary measurements.

FESRT: Functional Electrical Stimulation Row Training, SOC: Standard Of Care, Tg: triglyceride, Chol: Cholesterol, LDL: Low-density lipoprotein, HDL: High-density lipoprotein, HbA1C: Hemoglobin A1C, HOMA-IR: Homeostatic Model Assessment for Insulin Resistance, QUICKI: Quantitative insulin sensitivity check index.

Calculated BMRs for each group revealed a significant time and group interaction indicating that FESRT led to a significant increase in BMR of 53.7 kcal.d^-1^ (p = 0.006). However, both FESRT and AO training were associated with a greater BMR increase compared to WL (85.6 kcal.d^-1^, p = 0.001 and 59.2 kcal.d^-1^, p = 0.017 respectively), but only FESRT resulted in a significantly higher BMR than WL (FES: 1531.9 kcal.d^-1^, AO: 1511 kcal.d^-1^, WL: 1448.1 kcal.d^-1,^ p = 0.003).

## Discussion

This is the first study assessing the effects of FESRT on body composition in the sub-acute 24 months after SCI. We found that six months of high-intensity full-body exercise significantly increased exercise capacity and led to a tendency for lesser total body fat accrual and to significantly increased total and leg lean mass compared to SOC. Among FESRT and AO exercise, only FESRT led to significantly higher total lean mass compared to the wait list. Regular FESRT exercise during the subacute phase also attenuated BMD loss. Interestingly, the bone preserving effects were also significant after delayed training in those participants who crossed over to FESRT. Compared to SOC, participants in FESRT group had higher pelvis BMD and a modest tendency to greater total BMD. However, no effects were observed on cardiometabolic indices except for HbA1c in those who performed FESRT after SOC.

Full body exercise in SCI that engages paralyzed muscle through electrical stimulation has been proposed as a potentially effective method to alleviate the effects of immobility on body composition. The primary beneficial outcomes are increasing or maintaining lean mass and attenuating increases in body fat [16, 17]. Prior work suggests that six weeks of intensive FESRT and twelve weeks of neuromuscular electrical stimulation resistance training are relatively effective to increase lean mass and decrease percent body fat in individuals with chronic SCI [33, 34]. Similar work found that 12 weeks of FESRT in chronic SCI (time since injury: 20.9 ± 5 years) tends to reduce fat mass and significantly decrease plasma glucose [35]. In those with more acute injuries (24–35 months post SCI), locomotor training via epidural stimulation increases fat-free mass and attenuates body fat accrual [32].

Changes in body composition are most rapid and severe in the early stages after injury. Lean mass and muscle cross sectional area are rapidly decreasing while fat is rapidly increasing, leading to secondary effects on metabolism [1, 2, 4, 8, 9]. In fact, after SCI, leg lean mass substantially declines within the first 15 weeks with a mean decrease of 15% in the first year [46]. Our results indicate that in contrast to arms only exercise, FESRT significantly increases leg lean mass. Surprisingly, body composition changes with initial FESRT were more robust than those with 6 months of FESRT after SOC. This would suggest that early interventions may be most preventative of declines and is consistent with data suggesting that exercise of the paralyzed limbs during the first 12 weeks after injury results in paralyzed muscle hypertrophy associated with increased muscle cross-sectional area, power, and mass [30]. Our results also suggests significantly higher BMR after FESRT, indicating relatively lesser loss of metabolically active muscle mass. Increased BMR can prevent the imbalance between energy intake and expenditure that lead to obesity in SCI [47].

Prior research suggests visceral adipose tissue, making up to 6% of body fat mass, may be more than 50% higher in SCI patients compared to able bodied participants [5].VAT volume is associated with increased metabolic risk and relates proportionally to changes in BMI [5, 47]. Our results suggest relatively less VAT increases with whole body exercise in the subacute phase of SCI (Fig 2), hence it is possible that a longer FESRT exercise intervention could result in a bigger and more generalizable effect.

During the first year after SCI, bone mineral content decreases 4% per month in trabecular and 2% per month in cortical bone [46]. This leads to a rapid and significant bone loss of 45% in the pelvis and 25% in the legs [46]. It has been previously shown that those with a longer time since injury have relatively lower trabecular BMD and thickness and FESRT coupled with pharmacotherapy can counter the negative time‐dependent effects of SCI on bone density and microstructure [31]. Our current results indicate that early application of sufficient load to sub-lesional bones via FESRT without pharmacotherapy can have a significant effect in preserving BMD, specifically in the involved pelvic region compared to SOC. These findings are consistent with results of prior research that indicates FES resistance standing exercise early after SCI may counter the trend of decreasing BMD in the lower limbs [48].

Serum levels of low-density lipoprotein, high-density lipoprotein, total cholesterol, and triglycerides decrease acutely after SCI and gradually increase to normal levels during the first year [49]. Afterward, serum lipids gradually increase to high-risk levels which is associated with the extended inactivity and changes in body composition [50]. FESRT had no effect on cardiometabolic indices, except on HbA1c levels in SOC subjects who received a subsequent 6 months of this training. It may be that substantial metabolic risk-related changes do not occur in the early months after SCI [49], especially in a young and healthy population. Hence, exercise effects on cardiometabolic indices may not be expected. On the other hand, decreased HbA1c levels after delayed FESRT may suggest that FESRT would have preventative effects at the later stages of subacute SCI.

**Fig 4:**
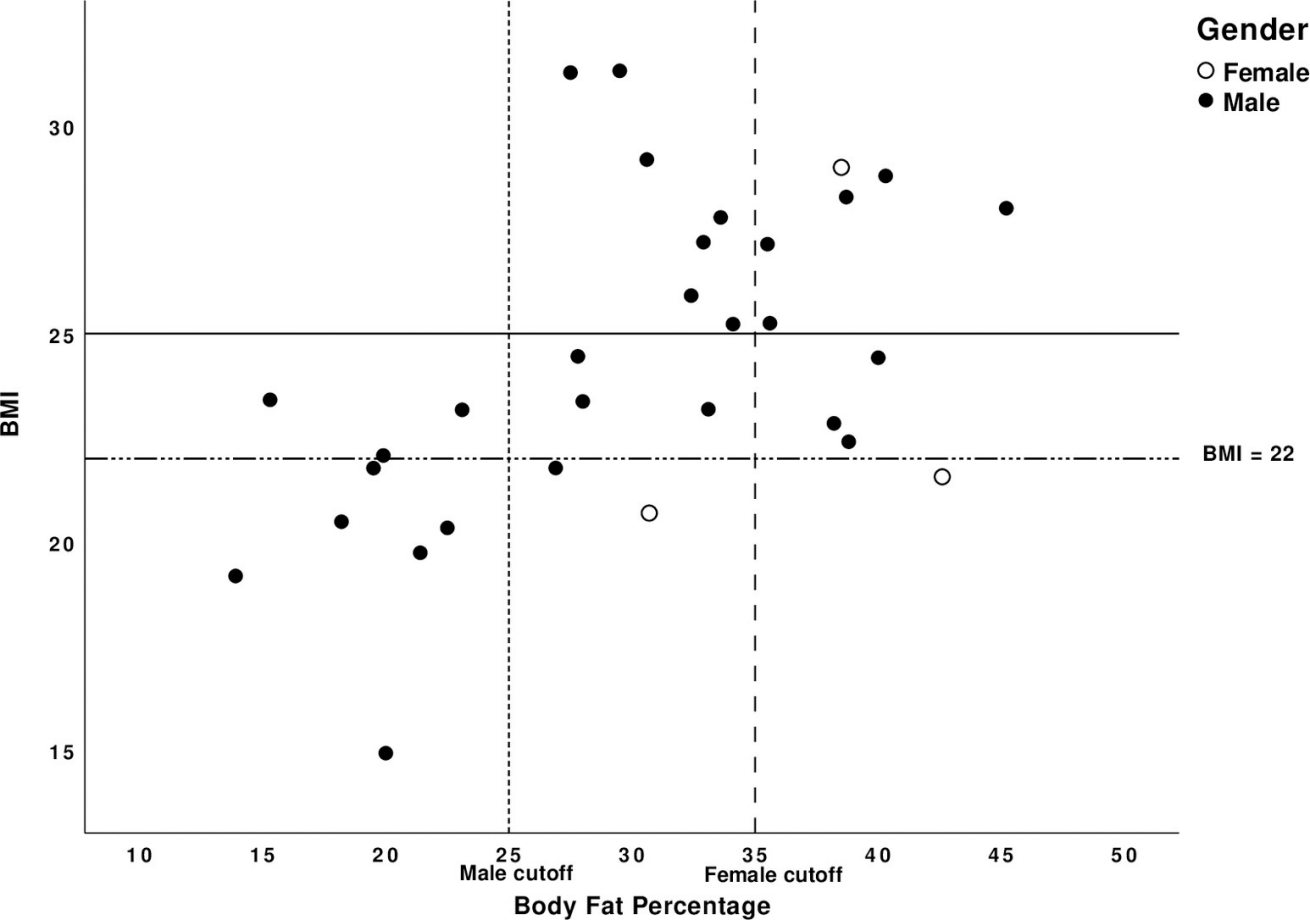
BMI and body fat percentage association. Scatter plot showing each participant’s initial BMI with matching calculated body fat percentage through DXA imaging. Normal population and spinal cord injury patients overweight BMI cut-off values of 25 and 22 are represented as horizontal lines respectively. Body fat percentage obesity cut off values of 25% (male) and 35% (female) are also represented as a vertical line.

One other facet of our data is insight to BMI and body fatness in SCI. Although a BMI over 25 is considered overweight based on CDC and WHO guidelines, Laughton et al. introduced the SCI-specific BMI cutoff ⩾22 kg.m^-2^ to indicate over fatness in this population. This was based on assessment in adults more than one year after SCI (14.7±10.5 years, range: 1–45 years) [51]. However, this value may not apply in the subacute phase of SCI prior to marked changes in body composition. Therefore we explored the relation of BMI to body fat prior to training in our participants. Fig 4 shows that though our participants were all in the subacute phase, the BMI cutoff was accurate to detect obesity. Indeed, the SCI-specific BMI cutoff had a relatively high sensitivity and specificity of 90% and 80% based on DXA derived body fat percentage. Hence, the SCI-specific BMI cutoff of 22  kg.m^-2^ appears to be appropriate across the entire span of time since injury.

The exclusion of older individuals and those with cardiovascular disease meant that our study population was in exclusively young healthy individuals. Thus, we encountered no safety issues, overuse injuries, or adverse events. Further studies are needed to determine the safety thresholds for other populations of individuals with SCI. The current study’s results are limited by the difficulty of retaining a large number of SCI patients within the subacute phase of injury for up to a year. In addition, as with all exercise intervention trials in humans, the ability to exercise train individuals is limited by compliance. Nonetheless, the results indicate that high intensity hybrid FES exercise can be effective in mitigating deleterious body composition changes in the sub-acute phase after SCI.

## Conclusion

In conclusion, this clinical trial suggests FESRT early after SCI can have substantial effects on preventing negative changes in body composition. Additionally, this exercise may counter the time dependent decrease of bone density and improve the delayed secondary metabolic alterations.

## Supporting information

S1 Checklist CONSORT 2010 checklist of information to include when reporting a randomised trial*. (DOC)

S1 File (DOCX)

## Abbreviations

SCI: Spinal Cord Injury
FES: functional electrical stimulation
RT: Row Training
SOC: Standard of Care
AO: Arms Only
WL: wait list
BMI: Body Mass Index
VO_2peak_: peak oxygen uptake
ASIA: American Spinal Injury Association
VO2: O2 consumption
VCO2: CO2 production
RER: respiratory exchange ratio)
VE: ventilation
DXA: dual x-ray absorptiometry
VAT: visceral adipose tissue
BMD: Bone Mineral Density
BMC: Bone Mineral Content
ANCOVA: Analysis of Covariance
BMR: Basal Metabolic Rate
Tg: triglyceride
Chol: Cholesterol
LDL: Low-density lipoprotein
HDL: High-density lipoprotein
HbA1C: Hemoglobin A1C
HOMA-IR: Homeostatic Model Assessment for Insulin Resistance
QUICKI: Quantitative insulin sensitivity check index
