# Supplementary material for: Effect of hybrid FES exercise on body composition during the sub-acute phase of spinal cord injury
Source: PLoS One. 2022 Jan 24;17(1):e0262864. doi: 10.1371/journal.pone.0262864 (PMC8786191; doi:10.1371/journal.pone.0262864)
Supplement: S1 File — (DOCX) [file pone.0262864.s002.docx]

Answer all questions accurately and completely in order to provide the PHRC with the relevant information to assess the risk-benefit ratio for the study. Do not leave sections blank.

# PRINCIPAL/OVERALL INVESTIGATOR

J. Andrew Taylor, PhD.

# PROTOCOL TITLE

Hybrid-FES Exercise to Prevent Cardiovascular Declines in Acute and Chronic SCI

# FUNDING

National Institutes of Health

# VERSION DATE

01/09/18

### SPECIFIC AIMS

Concisely state the objectives of the study and the hypothesis being tested.

**Specific Aim 1:** To document changes in body composition (visceral adiposity) and insulin sensitivity (homeostatic model assessment) resulting from FES-RT in comparison to a time (wait-list) control and to arms-only-RT. **Hypothesis:** FES-RT will mitigate against increased visceral adiposity and reduced insulin sensitivity occurring with acute SCI and that FES-RT will be more effective than arms-only-RT.

**Specific Aim 2:** To quantify changes in key indices of cardiovascular health (serum lipids) and control (arterial baroreflex gain) with FES-RT in comparison to a time control and to arms-only-RT.

**Hypothesis:** FES-RT will prevent the worsening lipid profile and compromised baroreflex function occurring with acute SCI and that FES-RT will be more effective than arms-only-RT.

**Specific Aim 3:** To characterize changes in myocardial structure (left ventricular wall thickness) and function (left ventricular diastolic velocity) with FES-RT in comparison to a time control and to arms-only-RT.

**Hypothesis:** FES-RT will counter the ventricular wall thickening and declining ventricular function occurring with acute SCI and that FES-RT will be more effective than arms-only-RT.

**Specific Aim 4:** To compare the magnitude of adaptations across a range of injury duration between acute and chronic SCI.

*We will explore the differences between acute and chronic SCI in all the outcome variables in aims 1,2 and 3.*

# BACKGROUND AND SIGNIFICANCE

Provide a brief paragraph summarizing prior experience important for understanding the proposed study and procedures.

Each year, 11,000 people suffer a spinal cord injury (SCI) in the U.S. In the first year after an SCI, there are profound declines in function, forming an underlying substrate for future cardiovascular morbidity and mortality. In fact, acquired cardiovascular disease (CVD) is an increasingly recognized consequence of SCI and is the leading cause of death in this population. Almost all risk factors are amplified in those with SCI;^2^ though incompletely understood, the almost 10-fold prevalence of CVD results at least in part from profound physiologic detraining resulting from motor impairment and immobility. Moreover,physical rehabilitation after SCI needs to move beyond the goal of maximizing independence to focus on maintenance of optimum health and fitness; thus, vigorous exercise of the paralyzed limbs may help achieve these objectives as well as reduce cardiovascular risk. However, effective interventions preventing acute declines that lead to cardiovascular compromise and increased risk in SCI are lacking.

An appropriate exercise intervention early after injury could prevent or lessen many of the acute declines that predispose those with SCI to a myriad of long-term health risks. However, exercise therapy for those with SCI is challenging and when employed, is typically limited to upper body activity. Recently, we developed a unique form of exercise for those with SCI that specifically mirrors exercise performed by those without SCI. Functional Electrical Stimulation Row Training (FES-RT) couples volitional arm and electrically controlled leg exercise. In contrast to other forms of exercise (arms only, FES leg cycling, or FES leg cycling with arm exercise), FES-RT is unique in that the non-innervated legs are integral for the performance of this whole body exercise. The full rowing stroke is produced by both the (stimulated) legs and arms, increasing the active muscle mass and resulting in a hemodynamic profile that produces the beneficial cardiac loading conditions of large muscle mass exercise. As such, FES-RT may be a safe and effective way to attenuate cardiovascular declines following SCI. Our preliminary work in chronic SCI has demonstrated the feasibility of FES-RT and suggests important attendant changes in cardiovascular health.^4^However, it is imperative that the positive impact of FES-RT be determined in those with acute SCI, because it is at the early stages of injury that future medical complications can be prevented. We will also study those with chronic SCI to provide a comparison of the magnitude of adaptations across a range of injury duration.

###### RESEARCH DESIGN AND METHODS

Briefly describe study design and anticipated enrollment, i.e., number of subjects to be enrolled by researchers study-wide and by Partners researchers. Provide a brief summary of the eligibility criteria (for example, age range, gender, medical condition). Include any local site restrictions, for example, “Enrollment at Partners will be limited to adults although the sponsor’s protocol is open to both children and adults.”

We will enroll 60 individuals to start study assessments within 3-12 months post SCI to obtain data on 50 individuals, forty percent of the subjects (N=20) will be randomized to immediately enroll in 6 months of FES-RT. Thirty percent (N=15) will be randomized to a wait-list to provide time control data from baseline to six months. A wait-list control group is routine in exercise studies because most volunteers are interested in participating in an exercise program. Hence, time controls are difficult to capture since many of those randomized to receive no exercise either drop from the study entirely, or end up pursuing some form of exercise on their own. Therefore, a vehicle for enrollment of a time control group that is acceptable to most volunteers is a wait-list. A 6 month time control will provide data on expected declines. Thirty percent (N=15) of subjects will be randomized to six months of arms-only-RT. This will provide data on a comparator that represents a form of arms-only exercise that would be available to those with SCI. These subjects will undergo 6 months of FES-RT after completing arms-only RT. This will enhance subject retention and provide data for secondary analysis. All volunteers will continue with regular physical rehabilitation except for modalities that overlap study interventions (e.g., FES-cycling).

To enhance subject recruitment we may enroll subjects into the study before they are 3-months post injury, most likely while they are still in-patients at Spaulding Rehabilitation Hospital. This will help subjects to schedule and plan for transportation for study assessments and training to begin when they reach 3-months post injury. No study assessments will be scheduled before 3-months from time of their SCI. Study participation will not interfere with scheduling of any normal inpatient therapy, nor will study participation take place during normal therapy. In-patients will need to be cleared to participate by their treating physician.

In addition we will enroll another 25 individuals with chronic SCI to serve as a comparator to the “Acute” FES-RT group. Subjects in the chronic group will be at least 1 year post spinal cord injury with adult onset of SCI(SCI occurrence after 18 years of age).

All subjects will 18-40 years of age with spinal cord injury (American Spinal Injury Association A, B, C) at the neurological level of C5-T12. Subjects will be medically stable (no treatment for DVT, no orthostasis, no spinal precautions or weight-bearing precautions associated with a healing long-bone fracture) and be able to tolerate the stimulation without dysreflexia. Dysreflexia in some participants is expected in the initial FES sessions, but only those in whom dysreflexia abates with repeat exposures will be included.

Participants will be excluded if they have: 1)Hypertension(blood pressure >140/90 mmHg), 2) significant arrhythmias, 3) coronary disease, 4) diabetes, 5) renal disease, 6) cancer, 7) epilepsy, 8) current use of cardioactive or antidepressant medications, 9) family history of significant arrhythmia or sudden cardiac death 10) orthostatic hypertension with symptomatic fall in blood pressure > 30 mmHg when upright 11) current grade 2 or greater pressure ulcers at relevant contact sites, 12) other neurological disease, 13) peripheral nerve compressions or rotator cuff tears that limit the ability to row, 14) history of bleeding disorder, 15) regular use of tobacco, 16) current deep venous thrombosis, 17)severe spasticity(that prevent rowing movements), 18) severe autonomic dysreflexia, 19)implanted electronic cardiac device (pace maker, cardiac defibrillator), 20) ventilator-dependent condition, 21)sensations that prevent the use of the electrical stimulation, 22) other medical contraindications to vigorous exercise

The American College of Sports Medicines Guidelines for Exercise Testing and Prescription 9^th^ edition will be followed as the standard of care along with the clinical specialists opinion (Drs. Stephanie Cho and Aaron Baggish) regarding any additional pre-participation screening needed prior to beginning this vigorous exercise training program.

Pregnant and nursing women are not eligible for this study because of the potential risk to the fetus from radiation. Women who are able to become pregnant must agree to use contraceptive methods while participating in the study. Acceptable contraceptive methods are: a) hormonal methods (birth control pills, patches, injections, vaginal ring or implants); b) barrier methods, (condom or diaphragm, with a foam, cream or gel spermicide); c) intrauterine device (IUD); or d) abstinence (no sex). Women are considered unable to become pregnant if they are menopausal and have not had a menstrual period for at least 1 year or if they have had a hysterectomy (surgical removal of your uterus and/or ovaries).

Briefly describe study procedures. Include any local site restrictions, for example, “Subjects enrolled at Partners will not participate in the pharmacokinetic portion of the study.” Describe study endpoints.

Volunteers who enroll in the study before 3-months from their injury date will have a partial screening visit in which informed consent and detailed health history will be obtained(all other assessments will be scheduled after they reach at least 3-month post SCI. Once recruited for the study volunteers (that are at least 3-months post injury) will visit the laboratory for a health screening session to further determine eligibility. This two-hour session may require 1 or 2 visits depending on the ability to schedule with the physician. It will include obtaining informed consent, detailed health history, a standard physical exam (ASIA exam and Ashworth Scale) performed by a physician, height and weight measurements, resting blood pressure, electrocardiogram (EKG), and FES-device test.

Note: Subjects may also have their echocardiogram test performed at the Heart Center at Massachusetts General Hospital (MGH) if the cardiologist can be scheduled for a more convenient time for them at MGH than at Spaulding Cambridge. We will pay for parking if they choose to go to MGH for their echocardiogram.

Note: If an ASIA exam and Ashworth Scale have previously been performed by a physician and results can be obtained, those medical records may be used and the physical exam will not be necessary during screening.

Subjects may also be asked to provide additional medical records or may need further testing to determine their eligibility and the safety of their participation in this program which includes regular vigorous exercise. These additional tests may or may not be covered by insurance. The subjects will be responsible for obtaining any additional testing and providing the program with medical records or results.

Approximately 40% of spinal cord injured women have amenorrhea for an average of 8 mos. Therefore, menstrual status will be tracked and explored as a potential covariate. A prospective menstrual diary will be collected for 3 months prior to each data acquisition time-point. For each monthly diary, women will mark the day of the month when bleeding occurred and rate the heavy). If no bleeding occurred during a particular month or the menstrual period, this will be indicated. Bleeding patterns will be used to define normal, irregular, or absent menstrual cycles.

**Study Groups and Training Protocols**

1)FES-RT Group(Acute and Chronic), 2) Arms-Only-RT Group, 3) Wait List Group

**1) FES-RT GROUP (Acute and Chronic)**

FES-strength training prior to FES-RT

To be able to FES-row, a certain level of leg muscle strength and endurance is essential; therefore, prior to FES-row training, the ability of the subject to perform 30 minutes of leg FES-induced extension will be evaluated. Small active electrodes will be placed over the motor points of the rectus femoris, vastus medialis vastus lateralis, biceps femoris and semitendinosos . A 4-channel Odstock stimulator will alternate stimulation between the hamstrings and the quadriceps while the subject is seated on their wheelchair or an exam table. The stimulus parameters will be set at 12 second period, with 6 second on-time per channel, without ramp and pulse width of 450ms at 50 Hz. The intensity of the stimulus to the quadriceps will be set at the level that produces full knee extension. If the knee does not fully extend (for example due to fatigue) the intensity of the stimulation will be increased. If fatigue does not occur prior to 30 minutes, the individual will advance to FES-rowing. If fatigue occurs prior to 30 minutes and at maximal intensity (approximately 100mA), the subject will engage in two weeks (3x/week) of strength training using this same protocol. Previous experience has indicated that this protocol is effective in enhancing muscle strength. Following two weeks of training, the strength and endurance capacity of the knee-extensor muscles will be evaluated and further training will be performed, if necessary, including the additional use of the QAA-9054 Total Power strength training machine. The QAA-9054 is a unique machine that provides the safety of accommodating resistance without the need of eccentric contractions. This type of resistance will not only allow the proper resistance to be safely placed on the knee extensors but also the knee flexors. Our past experience has shown us that the knee flexors may lag behind the knee extensors and prevent continuous FES-rowing as we can only increase the FES-stimulus to the knee flexors to cause a mild contraction during strength training. The Strength training will continue until the subjects are able to complete the above knee flexion-extension protocol for 30 minutes without rest. From past experience this may take between 2 and 12 weeks depending on how the subjects respond to the FES-strength training. Subjects will also be encouraged to begin arms only rowing during the initial FES-strength training period. This will be done at the same visits as the FES-strength training sessions. Participants will be seated on the rowing machine with the rowing seat locked in a fixed position and begin with short intervals of rowing at a moderate intensity using only their arms with intensity monitored by heart rate and blood pressure response and rating of perceived exertion. Subjects will be allowed to take and use the FES-devices at home after they have completed the first two weeks of training if they have showed adequate understanding of how to use the FES-devices properly. Subjects will perform strength training sessions at Spaulding Boston or Spaulding Cambridge as needed during home strength training. Subjects will also be given a logbook to help track their progress.

FES-RT

Subjects will begin with short intervals of FES-RT with rest intervals and/or arm only rowing intervals interspersed depending on their fitness level and the response of their legs to the FES (quite often three 5-minute intervals of alternating FES and arms-only rowing). The FES row system will optimize the contribution of the pre-trained muscle components of quadriceps and hamstrings. To maintain the proper training stimulus at the same relative intensity, a maximum FES-rowing test will be performed at baseline and after three months of training and training intensity will be re-determined. The goal is for each volunteer to achieve an exercise intensity of 75-85% maintained for a continuous 30 minutes performed three times each week.

Measurements of force produced at the foot and handle during FES-row training sessions will also be used to monitor training and possibly improve rowing technique.

###### Strength Training during FES-RT

After the first two weeks of FES-leg strength training (it may take longer depending on the subjects response, see above) and when the subjects have demonstrated the knowledge and ability of proper use of the stimulation unit they will use the stimulation unit at home to continue with leg strengthening concurrently on non FES-rowing days (~3 days/week, 30-60 minutes/session). This will be closely monitored, as they will also be FES-rowing three times per week at Spaulding Boston or Spaulding Cambridge.

**2) ARMS-ONLY-RT GROUP(Acute SCI only)**

The arms-only-RT group will commence exercise training upon enrollment. Training sessions will be three times per week for 26 weeks. To parallel the FES-RT, the initial training sessions will also consist of 6 sets of arms-only rowing for five minutes at 60% of VO2peak with a work-to-rest ratio of 2:1 and progress over the six months to an exercise intensity of 75-85% maintained for 30 minutes performed three times each week. The arms-only-RT group will also perform the FES-leg strength training protocol as described above during their six months of arms-only training. During this time they will not use the FES-device on the rowing ergometer. Strengthening the leg muscles during the arms-only period will lessen leg muscle atrophy and allow faster progress in FES-row training when they enter that phase of the study. A maximum arms-only rowing test will be performed after initial familiarization with arms-only-RT equipment (usually 2-3 sessions) and after three and six months of training. Training intensity will be determined with data obtained at the baseline and three month VO2max tests. At the end of six months of arms-only-RT subjects will begin the FES-RT as described above

**3) WAIT-LIST GROUP(Acute SCI only)**

The wait list group will not participate in row training for six months. A maximum arms-only rowing test will be performed immediately after enrollment, after initial familiarization with arms-only-RT equipment (usually 2-3 sessions) and will be repeated after three and six months (before FES-RT begins). The wait-list group will also perform the FES-leg strength training protocol as described above during the six months of wait-list period. During this time they will not use the FES-device on the rowing ergometer. Strengthening the leg muscles during the wait-list period will lessen leg muscle atrophy and allow faster progress in FES-row training when they enter that phase of the study. At the end of the six month wait period, subjects will begin the FES-RT as described above, (beginning with FES-strength training prior to FES-row training).

Alternate Training Sites:

Community Rowing Inc. as an additional training site

After subjects have completed screening and initial leg muscle strengthening, and have advanced in their FES-RT to the point at which they demonstrate sufficient knowledge and skill level at performing the FES-RT or arms-only-RT they may begin training at our alternate training site, Community Rowing Inc (CRI).There will be a SRH staff member on-site at CRI to supervise all FES-row or arms-only row training sessions. Also, only subjects who have demonstrated the ability to transfer onto the rower safely with minimal assistance will be allowed to perform the FES-RT or arms-only-RT at CRI. (There is not a lift system available at CRI.) All Laboratory visits will continue to be performed at Spaulding Hospital Cambridge or Spaulding Charlestown.

Home Rowing as an additional training site

Subjects who own an adapted rowing machine may perform some or all of their row training at home. Home rowing will only be allowed after subjects have completed screening and have advanced in their FES-RT or arms-only-RT to the point in which they can demonstrate sufficient knowledge and skill level in the use of the FES-unit and /or the adapted rowing machine and understanding of their individualized exercise program. All subjects must also demonstrate transfer competency on and off the rower with use of the assistance that they will have available at home before being allowed to begin home rowing. All Laboratory visits will continue to be performed at Spaulding Hospital Cambridge or Spaulding Charlestown.

Notes:

There is no limit or specific requirement for training sessions that must be attended to continue participating in this protocol. Subjects, in all training groups, will be encouraged by study staff to attend three training sessions per week. Un-reported missed training session will be followed up with a phone call to document why the training session was missed and to encourage prompt return to training sessions as soon as possible.

Subjects who enroll before 3-months from their date of SCI will be given the opportunity to participate in the FES-strength training during the period before study assessments begin after they reach 3-months post SCI. This will lessen leg muscle atrophy and allow faster progress in FES-row training when they enter that phase of the study.

**Study Assessments**

All Assessments will be performed at baseline and after three and six months for each study group except the DXA measurements which will only be performed at baseline and after six months. The Arms-only-RT and Wait-list groups will immediately enter baseline FES-RT protocol after completing all study procedures required in their initial group. Their six month lab visit and DXA scan data will also be used as their baseline FES-RT measures. Subjects may be asked to repeat any of the study assessments if there is difficulty obtaining results that meet our quality standards or there are equipment issues that arise.

Subjects that take longer than eight weeks to progress to performing their baseline FES-VO2max test will perform an extra assessment around the time of their baseline FES-VO2max test to account for changes that may occur over that time period. This extra study session will consist of: height, weight and circumference measures, blood samples, health questionnaires, pulmonary function tests, resting cardiovascular hemodynamics and neck suction-barorefex testing. This extra testing session will take about 2-hours and can be done on a normal training day.

Study Procedure Time Line:

|  | Baseline | 3-Month | 6-Month | Baseline-RT | 3-Month-RT | 6- Month-RT |
| --- | --- | --- | --- | --- | --- | --- |
| FES-RT  Acute and Chronic | FES-VO2max  AO-VO2max  Lab Visit  DXA Scan | FES-VO2max  Lab Visit | FES-VO2max  AO-VO2max  Lab Visit  DXA Scan | N/A | N/A | N/A |
| AO-RT  Acute | AO-VO2max  Lab Visit  DXA Scan | AO-VO2max  Lab Visit | AO-VO2max  Lab Visit  DXA Scan | FES-VO2max | FES-VO2max  Lab Visit | FES-VO2max  Lab Visit  DXA Scan |
| Wait list  Acute | AO-Vo2max  Lab Visit  DXA Scan | AO-VO2max  Lab Visit | AO-VO2max  Lab Visit  DXA Scan | FES-VO2max | FES-VO2max  Lab Visit | FES-VO2max  Lab Visit  DXA Scan |

Measurements and Testing Protocols:

VO2max Testing

For subjects in the arms-only-RT and FES-RT groups VO2max testing will be performed on a day scheduled for regular training. On-line computer-assisted open circuit spirometry will be used to determine O_2_ consumption, CO_2_ production, and respiratory exchange ratio. Expired O_2_ and CO_2_ gas fractions will measured with a paramagnetic O_2_ and infrared CO_2_ analyzers. Ventilation will be measured via a Hans Rudolph 3813 pneumotachograph. Subjects will perform FES-rowing or arms-only-rowing while increasing power output every one to two minutes until volitional exhaustion. Immediately after FES-rowing or arms-only-rowing has stopped at the end of the VO2max test, subjects will have a finger tip pricked with a safety lancet in order to get a drop of blood that will be used to measure peak lactate levels in whole blood. Peak lactate levels will be used to evaluate exercise intensity as well as to monitor training status. To ensure attainment of maximal exercise capacity, at least three of the following criteria will be met: 1) O_2_ consumption plateaus despite increasing workload, 2) respiratory exchange ratio equals or exceeds 1.10 at end exercise, 3) 85% age-predicted maximal heart rate is achieved, and 4) perceived exertion is rated at least 17 on the Borg scale of 6-20.

Lab Visit(s)

All lab studies will be performed in the morning. All subjects will be instructed to abstain from vigorous exercise for two days prior to each study to avoid autonomic and neuroendocrine effects of exercise. In addition, subjects will refrain from caffeine and alcohol for the previous 24 hrs, and be studied after a 12 hr fast.

Neck Suction- Baroreflex Testing:

We will rely on neck pressure and neck suction to evaluate carotid sinus baroreceptor function at rest and during a variety of conditions. This technique has been employed to safely study cardiovascular physiology in human since the 1950’s. The subject’s neck is sealed by a neck collar connected to a computer-controlled bellows (pressure/vacuum pump). The neck collar is cushioned with an airtight silicon rubber bladder that is independently sealed and positioned against the contour of the anterior lateral neck. The bellows is used to increase or decrease pressure inside the neck collar, effectively compressing or distending the carotid sinus in the neck and causing reflex changes in heart rate and muscle sympathetic activity. However, these responses are evanescent since aortic baroreceptors are not affected; therefore heart rate and sympathetic activity (and sometimes blood pressure) are changed for only a few beats with any level of stimulus. Carotid baroreceptor reflex responses will be elicited during held expiration by application of an external neck pressure and suction sequence. Subjects will hold a normal end-expiratory volume, which triggers an external neck pressure increase of +40 mmHg for 4 heart beats and subsequent, serial R-wave triggered reductions of 15 mmHg to a pressure of -65 mmHg. This sequence results in stepwise initial carotid sinus compression followed by distension, evoking stepwise reflex changes in heart rate and sympathetic activity. For each baroreflex testing, this procedure is repeated seven times to provide an averaged reproducible carotid baroreceptor reflex response relation.

*Echocardiography.* We will use a portable, commercially available system (Vivid-I, GE Healthcare, Milwaukee, Wis.), for cardiac characterization. Images will be obtained after 20 minutes of rest. Conventional 2-dimensional, Doppler, and color tissue-Doppler imaging from standard parasternal, apical, and subcostal positions will be performed. All data will be stored digitally for post-study off-line data analysis (EchoPac, Version 6.5, GE Healthcare). Left ventricular and atrial cardiac chamber volumes will be calculated using the modified Simpsons technique. LV mass will be calculated using the area-length method. Relative wall thickness, an index of LV geometry, will be defined as: [interventricular septal thickness (mm) + posterior wall thickness (mm)] / LV internal end-diastolic diameter (mm). Myocardial tissue velocities (diastolic function) will be measured off-line from 2D color-coded tissue Doppler images and reported as the average of three consecutive cardiac cycles. Strain, strain rate, and LV rotational (systolic function) assessment will be performed by speckled tracking analysis. Peak global strain will be calculated as the average strain in the basal, mid, and apical LV as measured in the apical 4-chamber view. Atrial volumes will be calculated using the bi-plane area-length method. Right ventricular diastolic area, systolic area, fractional area change, tissue velocities will be measured. Measurements will be indexed for body surface area when appropriate.

Note: As of 1/1/19 we will no longer be able to measure any further echocardiogram tests in this protocol.

Resting Cardiovascular Hemodynamics: Heart rate and blood pressures will be measured after a 15-minute period of quiet supine rest. Heart rate will be measured by a standard 12-lead ECG and blood pressure by an automated sphygmomanometer. There will be a short period of time paced breathing in which subjects follow an audio cue to breath at a rate of 15 breaths per minute.

Pulmonary Function Tests: Spirometry will be used to measure lung function, specifically the measurement of the amount (volume) and/or speed (flow) of air that can be inhaled and exhaled. Specifically, Forced Volume Vital Capacity (FVC) and Maximum Voluntary Ventilation (MVV) tests will be administered.

Height, Weight and Circumferences: Weight will be measured on a scale. Height will be measured in the supine position with a tape measure. Circumferences of the waist, hip, abdomen and thigh will be measured with a tape measure in the supine position.

Blood Samples: 70 ml of blood via venipuncture will be taken. Standard assays will include measures of insulin sensitivity and serum lipids. The homeostasis model assessment (HOMA) of insulin resistance, the quantitative insulin check index (QUICKI) and the serum insulin-like growth factor binding protein-1 (IGFBP-1) will be assessed. Plasma total cholesterol, low density cholesterol, high density APOa lipids, and triglycerides will also be measured. Other assays will include, testosterone(males only), CBC, C-reactive Protein, hemoglobin A1C, prealbumin and zinc.

ASIA and Ashworth exams: Standard physical exams will be performed by a physician to categorize injury level and degree of spasticity.

*Note: The ASIA and Ashworth exams will only be performed at Screening Visit 1 and 6-month and 12-month time points.*

Health Questionnaires: The PROMIS 57 questionnaire, the Promis Cognitive Short Form, the Patient Health Questionnaire (PHQ-9) the NIH Toolbox Meaning and Purpose Short Form, the SCI Exercise Self Efficacy Scale(ESES) and the Craig Handicap Assessment and Reporting Technique Short Form(CHART-SF) will be used to assess changes in subjects views on their physical, psychological and social well-being.

DXA Scan(s)

A licensed radiology technician will perform all DXA scans. We will use a 5th generation GE Healthcare iDXA dual x-ray absorptiometry (DXA) scanner with enCore configuration version 12.3. For regional fat measurements, the DXA software can be used to define standard regions that will allow comparability of measurements throughout the study. DXA-derived visceral adipose tissue will be measured from a 5 cm slice placed across the entire abdomen above the iliac crest at the 4th lumbar vertebrae. Although DXA measures both visceral and subcutaneous fat, on each side of the abdominal cavity DXA can directly measure subcutaneous fat. The location of the abdominal cavity is detected by the gray scale change as the tissue contains lesser fat due to abdominal wall muscles. Subcutaneous fat over the visceral cavity is estimated from the DXA measurement of subcutaneous fat on each side of the abdominal cavity. The total estimated subcutaneous fat is subtracted from the total abdominal fat for DXA-derived visceral adipose tissue. This approach has been shown to performed as well as a clinical read from a CT scan. The precision of regional fat measures is ±1%.

Note: If a DXA scan has recently been performed at Spaulding Rehabilitation Hospital and the results can be obtained, those medical records may be used and the baseline DXA scan will not be necessary.

For studies involving treatment or diagnosis, provide information about standard of care at Partners (e.g., BWH, MGH) and indicate how the study procedures differ from standard care. Provide information on available alternative treatments, procedures, or methods of diagnosis.

This program does not involve treatment or diagnosis. There are no alternative treatments or procedures for this study.

Describe how risks to subjects are minimized, for example, by using procedures which are consistent with sound research design and which do not unnecessarily expose subjects to risk or by using procedures already being performed on the subject for diagnostic or treatment purposes.

Risks to participants will be minimized by following the Spaulding Rehabilitation Hospital Policy and Procedure: General Safety Precautions and Procedures for the Conduct of Human Research Guidelines that has been approved by the SRH Professional Staff Executive Committee and the SRH HRC. During exercise testing and training a small percentage of subjects may develop myocardial ischemia. Other possible risks include abnormal blood pressure responses, fainting, arrhythmias, stroke, heart attack or even death. In reality, the relative risk for any of these developments is likely very small.

Describe explicitly the methods for ensuring the safety of subjects. Provide objective criteria for removing a subject from the study, for example, objective criteria for worsening disease/lack of improvement and/or unacceptable adverse events. The inclusion of objective drop criteria is especially important in studies designed with placebo control groups.

The study staff has made every effort to recognize risks and ensure the safety of program participants. All participants will be asked the “Pre-Study Screening” questions before every visit. The Cardiovascular Research Laboratory Medical Emergency Safety Plan will be followed in the case of an adverse medical event. Adverse events will be handled by immediately stopping any FES-stimulus, or exercise and monitoring the subject (including signs, symptoms, and blood pressure) as necessary, with immediate medical assistance available through Spaulding Charlestown or Spaulding Cambridge emergency procedures (i.e. calling the Stat Team or Code Team) if necessary.

# FORESEEABLE RISKS AND DISCOMFORTS

Provide a brief description of any foreseeable risks and discomforts to subjects. Include those related to drugs/devices/procedures being studied and/or administered/performed solely for research purposes. In addition, include psychosocial risks, and risks related to privacy and confidentiality. When applicable, describe risks to a developing fetus or nursing infant.

Moderate risks are associated with exercise testing and exercise training. Some discomfort and feeling of fatigue will be experienced during these tests. There are other risks associated with these tests including abnormal blood pressure responses, fainting, irregular, fast or slow heart rhythm, and in rare instances, heart attack, stroke, or death. Wearing the mouthpiece during the VO2max testing may cause feelings of claustrophobia. Exercise training may induce some muscle or joint discomfort when beginning an exercise program and could result in tendonitis and/or musculoskeletal overuse injuries over time. The electrical stimulation unit can cause tingling, pins and needles sensations on the skin, skin irritation, increased muscle spasticity and autonomic dysreflexia (signs include: headache, nausea, rise in blood pressure, sweating, and goosebumps). There is some discomfort with venipuncture and the lancet used on the finger for lactate measurement and there is the possibility of swelling and bruising at the site(s), there is also a slight chance that individuals become dizzy and even faint. The neck suction/pressure collar may cause some discomfort and a mild choking feeling which resolves quickly when pressure or suction is turned off. Subjects may feel lightheaded or short of breath during the pulmonary function tests, there is also a slight chance that individuals become dizzy and even faint.

The risk of DXA scanning is minimal. Participants in this study will be exposed to radiation from two or three DXA scans of the whole body depending on study group. The total amount of radiation that will be received from participation in this study is equal to a whole body exposure of less than 0.26 milliSierverts (mSv). For comparison, the average person in the United States receives a radiation exposure of 3.6 mSv per year from natural background sources (the earth and the sky). The dose from participation in this research study is about the same as what a person would normally receive in less than one month from these natural sources. Scientists disagree on whether radiation doses at these low levels are harmful. A possible effect that could occur at doses associated with this study is a slight increase in the risk of developing cancer later in life. Women of childbearing potential will be given a urine or serum pregnancy test before any DXA scan and not scanned if they are pregnant.

There are no known risks associated with the EKG or the echocardiogram. A subject must remove his or her shirt and have sticky patches applied to the hest for the electrocardiogram (EKG) and have gel applied for the echocardiogram. In some cases, chest hair may have to be shaved where the EKG patches are placed. If a structural abnormality is identified on an EKG or echo, the subject would be given this information and referred to his or her own doctor for further evaluation.

**EXPECTED BENEFITS**

# Describe both the expected benefits to individual subjects participating in the research and the importance of the knowledge that may reasonably be expected to result from the study. Provide a brief, realistic summary of potential benefits to subjects, for example, “It is hoped that the treatment will result in a partial reduction in tumor size in at least 25% of the enrolled subjects.” Indicate how the results of the study will benefit future patients with the disease/condition being studied and/or society, e.g., through increased knowledge of human physiology or behavior, improved safety, or technological advances.

Volunteers will have the opportunity to participate in a supervised exercise program, which may improve their overall cardiovascular fitness and endurance during activities of daily living as well as decrease risk of chronic disease.

**EQUITABLE SELECTION OF SUBJECTS**

The risks and benefits of the research must be fairly distributed among the populations that stand to benefit from it. No group of persons, for example, men, women, pregnant women, children, and minorities, should be categorically excluded from the research without a good scientific or ethical reason to do so. Please provide the basis for concluding that the study population is representative of the population that stands to potentially benefit from this research.

Program exclusion criteria are based mainly on health status. Subjects must be medically stable and meet the eligibility criteria for this study.

When people who do not speak English are excluded from participation in the research, provide the scientific rationale for doing so. Individuals who do not speak English should not be denied participation in research simply because it is inconvenient to translate the consent form in different languages and to have an interpreter present.

People who do not speak English will not be excluded from participation in this study.

For guidance, refer to the following Partners policy:

Obtaining and Documenting Informed Consent of Subjects who do not Speak English

[**http://healthcare.partners.org/phsirb/nonengco.htm**](http://healthcare.partners.org/phsirb/nonengco.htm)

**RECRUITMENT PROCEDURES**

Explain in detail the specific methodology that will be used to recruit subjects. Specifically address how, when, where and by whom subjects will be identified and approached about participation. Include any specific recruitment methods used to enhance recruitment of women and minorities.

We will rely mostly on word of mouth and referrals from the spinal cord injury program at SRH to enroll participants in this program. We also have a program information sheet and recruitment letters that may be sent to potential subjects. Once a potential subject expresses interest in the study they will be contacted either in person or by phone call to go over the Participant Phone Screening Form. If an in-patient at Spaulding Rehabilitation Hospital wants to participate in the study their treating physician will need to sign a clearance form before informed consent is obtained.

Provide details of remuneration, when applicable. Even when subjects may derive medical benefit from participation, it is often the case that extra hospital visits, meals at the hospital, parking fees or other inconveniences will result in additional out-of-pocket expenses related to study participation. Investigators may wish to consider providing reimbursement for such expenses when funding is available

Participants will not receive monetary remuneration for this study.

For guidance, refer to the following Partners policies:

Recruitment of Research Subjects

[**http://healthcare.partners.org/phsirb/recruit.htm**](http://healthcare.partners.org/phsirb/recruit.htm)

Guidelines for Advertisements for Recruiting Subjects

[**http://healthcare.partners.org/phsirb/advert.htm**](http://healthcare.partners.org/phsirb/advert.htm)

Remuneration for Research Subjects

[**http://healthcare.partners.org/phsirb/remun.htm**](http://healthcare.partners.org/phsirb/remun.htm)

#### CONSENT PROCEDURES

Explain in detail how, when, where, and by whom consent is obtained, and the timing of consent (i.e., how long subjects will be given to consider participation). For most studies involving more than minimal risk and all studies involving investigational drugs/devices, a licensed physician investigator must obtain informed consent. When subjects are to be enrolled from among the investigators’ own patients, describe how the potential for coercion will be avoided.

Research study staff will obtain volunteer consent in accordance with guidelines established by the Institutional Review Board during the first visit to the ExPD site. Participants will be sent the consent form at least 48-hours in advance so they have ample time to read and ask questions. Volunteers are encouraged to ask questions and are reminded that participation is strictly voluntary and will not affect their current or future care at Spaulding Rehabilitation Hospital or any of its affiliates.

NOTE: When subjects are unable to give consent due to age (minors) or impaired decision-making capacity, complete the forms for Research Involving Children as Subjects of Research and/or Research Involving Individuals with Impaired Decision-making Capacity, available on the New Submissions page on the PHRC website:

[**http://healthcare.partners.org/phsirb/newapp.htm#Newapp**](http://healthcare.partners.org/phsirb/newapp.htm#Newapp)

For guidance, refer to the following Partners policy:

Informed Consent of Research Subjects

[**http://healthcare.partners.org/phsirb/infcons.htm**](http://healthcare.partners.org/phsirb/infcons.htm)

## DATA AND SAFETY MONITORING

Describe the plan for monitoring the data to ensure the safety of subjects. The plan should include a brief description of (1) the safety and/or efficacy data that will be reviewed; (2) the planned frequency of review; and (3) who will be responsible for this review and for determining whether the research should be altered or stopped. Include a brief description of any stopping rules for the study, when appropriate. Depending upon the risk, size and complexity of the study, the investigator, an expert group, an independent Data and Safety Monitoring Board (DSMB) or others might be assigned primary responsibility for this monitoring activity.

NOTE: Regardless of data and safety monitoring plans by the sponsor or others, the principal investigator is ultimately responsible for protecting the rights, safety, and welfare of subjects under his/her care.

Data will be monitored throughout the duration of the program to ensure the safety of subjects. This will include the evaluation of resting ECG, blood work blood pressure, questionnaires and maximal exercise testing. The principal investigator will review all abnormal findings during the study.

Describe the plan to be followed by the Principal Investigator/study staff for review of adverse events experienced by subjects under his/her care, and when applicable, for review of sponsor safety reports and DSMB reports. Describe the plan for reporting adverse events to the sponsor and the Partners’ IRB and, when applicable, for submitting sponsor safety reports and DSMB reports to the Partners’ IRBs. When the investigator is also the sponsor of the IND/IDE, include the plan for reporting of adverse events to the FDA and, when applicable, to investigators at other sites.

NOTE: In addition to the adverse event reporting requirements of the sponsor, the principal investigator must follow the Partners Human Research Committee guidelines for Adverse Event Reporting

Serious adverse events will be reported to the IRB via phone, email, or fax. The principal investigator will follow this with a full written report using the PHRC Adverse Event Form within 10 working days. If a mild or moderate adverse event occurs that is definitely, probably, or possibly related to the study, a written report will be sent to the IRB within 20 working days. All other events will be summarized in a progress report at continuing review.

## MONITORING AND QUALITY ASSURANCE

Describe the plan to be followed by the principal investigator/study staff to monitor and assure the validity and integrity of the data and adherence to the IRB-approved protocol. Specify who will be responsible for monitoring, and the planned frequency of monitoring. For example, specify who will review the accuracy and completeness of case report form entries, source documents, and informed consent.

NOTE: Regardless of monitoring plans by the sponsor or others, the principal investigator is ultimately responsible for ensuring that the study is conducted at his/her investigative site in accordance with the IRB-approved protocol, and applicable regulations and requirements of the IRB.

The program director will be responsible for monitoring the completeness of all data and source documents. The Principal Investigator will monitor the informed consent procedures in accordance with the Informed Consent Compliance Checklist of Partners HealthCare Systems HRQIP. The subject’s data/protocol adherence will be monitored by the research coordinator at each step in the study including. Checklists and note pages are used to note any deviations or omissions from the protocols. Any clinical/health related issues would be immediately presented to the subject (i.e. abnormal EKG, blood chemistries, etc) to determine appropriate notification (i.e. current physician or appropriate specialist). Based on the seriousness of the situation Dr. Schwartz may be contacted to provide clinical guidance on the appropriate course of action as per the Cardiovascular Research Laboratory Medical Emergency Safety Plan.

For guidance, refer to the following Partners policies:

##### Data and Safety Monitoring Plans and Quality Assurance

[**http://healthcare.partners.org/phsirb/datasafe.htm**](http://healthcare.partners.org/phsirb/datasafe.htm)

Adverse Event Reporting Guidelines

**<http://healthcare.partners.org/phsirb/adverse_events.htm>**

# PRIVACY AND CONFIDENTIALITY

Describe methods used to protect the privacy of subjects and maintain confidentiality of data collected. This typically includes such practices as substituting codes for names and/or medical record numbers; removing face sheets or other identifiers from completed surveys/questionnaires; proper disposal of printed computer data; limited access to study data; use of password-protected computer databases; training for research staff on the importance of confidentiality of data, and storing research records in a secure location.

NOTE: Additional measures, such as obtaining a Certificate of Confidentiality, should be considered and are strongly encouraged when the research involves the collection of sensitive data, such as sexual, criminal or illegal behaviors.

Maintaining research volunteers’ privacy and keeping personal identifiers confidential is important to the study staff. All research activity including subject screening and data collection will be performed and stored at Spaulding Hospital Cambridge and Spaulding Hospital Charlestown. Subject names, contact information, health history, and other information that can be traced back to the subject will be kept separately from data collected for the study. Personal information will be kept in a locked office and away from data. Collected data will have the subject’s identification code and some computer software used to collect data will have the date and time marked on the file. Protocol sheets used during data collection will only have the subject’s ID. The exercise test will have a subject ID and may contain other pertinent information needed to clinically evaluate the test, such as age, medications, gender, and disease status. Blood and urine samples will be analyzed by Quest Diagnostics and will have the subject’s ID and birth date used for identifiers. Quest Diagnostics will send lab results to a secure fax machine located within the Cardiovascular Research Laboratory. Spaulding Hospital certifies key personnel have completed education on the use of human subjects in compliance with NIH regulations.

SENDING SPECIMENS/DATA TO RESEARCH COLLABORATORS OUTSIDE PARTNERS

Specimens or data collected by Partners investigators will be sent to research collaborators outside Partners, indicate to whom specimens/data will be sent, what information will be sent, and whether the specimens/data will contain identifiers that could be used by the outside collaborators to link the specimens/data to individual subjects.

Blood and urine specimens will be sent to Quest Diagnostics for analysis. Blood and urine specimens will contain a study identification number with date of birth used for identifier. Quest Diagnostics will send lab results to a secure fax machine located within the Cardiovascular Research Laboratory.

Specifically address whether specimens/data will be stored at collaborating sites outside Partners for future use not described in the protocol. Include whether subjects can withdraw their specimens/data, and how they would do so. When appropriate, submit documentation of IRB approval from the recipient institution.

Specimens/data will not be stored at collaborating sites outside of Partners for future use not described in the protocol.

# RECEIVING SPECIMENS/DATA FROM RESEARCH COLLABORATORS OUTSIDE PARTNERS

When specimens or data collected by research collaborators outside Partners will be sent to Partners investigators, indicate from where the specimens/data will be obtained and whether the specimens/data will contain identifiers that could be used by Partners investigators to link the specimens/data to individual subjects. When appropriate, submit documentation of IRB approval and a copy of the IRB-approved consent form from the institution where the specimens/data were collected.

Specimens or data collected by research collaborators outside Partners will not be sent to Partners investigators.
